# Sleep Quality and Compliance to Medical Therapy Among Hemodialysis Patients With Moderate-to-Severe Depression: A Cross-Sectional Study

**DOI:** 10.7759/cureus.13477

**Published:** 2021-02-21

**Authors:** Mehwish Kaneez, Syed Muhammad Jawad Zaidi, Abdullah Bin Zubair, Muhammad Rehan, Ahtisham Hassan, Zoya Sarwar, Aisha Bibi, Mahnoor Azhar, Kinza Kinza, Muzammil Sabir

**Affiliations:** 1 Internal Medicine, Rawalpindi Medical University, Rawalpindi, PAK; 2 Internal Medicine, Rashid Latif Medical College, Lahore, PAK; 3 Internal Medicine, Arif Memorial Teaching Hospital, Lahore, PAK; 4 Internal Medicine, King Edward Medical University, Lahore, PAK; 5 Psychiatry, Rashid Latif Medical College, Lahore, PAK

**Keywords:** end-stage renal disease (esrd), depression, sleep quality, compliance

## Abstract

Background

Depression is a fairly common finding among end-stage renal disease (ESRD) patients and is an independent risk factor for morbidity and mortality. The psychiatric manifestations of the disease may affect their compliance to medications and alter sleep quality that is often overlooked. This translates into poor quality of life and poorer disease prognosis. Our study aims to assess the prevalence of depression and its association with compliance to medical therapy and sleep quality among ESRD patients on hemodialysis.

Methodology

In this cross-sectional study, a total of 288 hemodialysis patients with a confirmed diagnosis of ESRD were evaluated for depression using Patient Health Questionnaire-9 (PHQ-9) scale. Only patients with moderate-to-severe depressive symptoms on PHQ-9 were further evaluated for sleep quality and compliance to medications using Pittsburgh Sleep Quality Index (PSQI) and Drug Attitude Inventory-10 (DAI-10), respectively. The characteristics of ESRD patients with depression were also assessed. Median PHQ-9, DAI-10, and PSQI scores were calculated, and the correlation between study variables was assessed using Spearman’s correlation.

Results

Of the 288 included participants, 188 (65.27%) had depression as evaluated via PHQ-9. Of these 188 patients, 114 were males while 74 were females. A total of 113 (60.01%) of depressed patients had poor compliance to medication while 137 (72.87%) patients had poor sleep quality. Higher PHQ-9 scores were positively correlated with disease duration, dialysis years, and time between diagnosis and therapy (r = 0.41, 0.39, and 0.43, respectively) and negatively with marital and employment status (r = -0.32 and -0.49, respectively). Spearman’s correlation showed a significant negative correlation of PHQ-9 scores with DAI-10 scores but a significant positive correlation with PSQI scores. The correlation between DAI-10 and PSQI was a significant negative correlation.

Conclusions

This study indicated a high prevalence of moderate-to-severe depression among ESRD patients on hemodialysis. Poor sleep quality and non-adherence to medications are frequent among ESRD patients with depression. These psychiatric components must be considered to optimize medical treatment and improve the quality of life in this subset of patients. More studies should be conducted to assess the risk factors of depression in patients with ESRD.

## Introduction

End-stage renal disease (ESRD) is a condition in which kidneys gradually and progressively fail to perform their metabolic, endocrine, and other regulatory functions [1]. Globally, the prevalence of ESRD is continuously increasing due to the high incidence of obesity, diabetes, and hypertension, which signifies its high disease burden, especially in developing countries [2,3]. Patients ultimately require frequent hemodialysis depending upon the disease stage for their survival [3]. Additionally, medical therapy with anti-diabetics and anti-hypertensives also plays a major role in improving the overall prognosis of the disease [4]. Non-compliance with medicines increases the need for frequent dialysis and hospital visits causing a vicious cycle of financial and psychological stress, which deteriorates the physical and mental health of such patients contributing to increased risk of mortality [5].

Frequent hemodialysis, fear of severe complications, financial burden, and limitations in daily life lead to severe mental effects such as depression [6]. Depression is regarded as a well-established psychiatric outcome of ESRD, and the literature reports an increased mortality rate among such patients [7]. The onset of depression has many important clinical implications along with associated psychological stress which eventually leads to hopelessness among ESRD patients and adversely affects their quality of life and disease state [8]. These patients then have low motivation to exercise, observe dietary control, and adhere to medications, which then translates into a poor disease prognosis and frequent hemodialysis in this subset of the population [9].

The dilemma is the underdiagnosis of the psychiatric components of ESRD. Lack of sufficient physician-patient interaction and medical management without necessary psychiatric consultation contributes to increased prevalence of depression in ESRD patients, which ultimately leads to dismal disease outcomes [10]. The literature shows that the underdiagnosis of depression in ESRD, particularly in developing countries, is contributing to more dismal disease outcomes [11]. Thus, to allow for effective patient management according to the biopsychosocial model [12], there is an urgent need to assess the prevalence of depression in ESRD so that optimal medical care is provided to such patients.

Sleep disturbance has been reported in up to 80% of patients undergoing hemodialysis due to kidney failure by many studies [13,14]. It is an established complication of ESRD which deteriorates the quality of life [14]. Moreover, the exact etiology of ESRD-associated sleep disturbance remains unknown. Our study hypothesizes that the underreported prevalence and effects of depression in ESRD patients might contribute to their sleep abnormalities.

This study aims to report the prevalence of moderate-to-severe depression among ESRD patients. We also aim to assess the correlation of sleep quality and compliance to medications with depression among patients with moderate-to-severe depression. Furthermore, we aim to identify their possible associations with various patient-related parameters. Because compliance with medical therapy affects disease prognosis, the results of this study may help to identify a patient population that is at a higher risk for non-compliance and hence poor outcomes among hemodialysis patients. This will aid healthcare professionals to timely manage these high-risk patients so that the medical care provided to them can be optimized.

## Materials and methods

This cross-sectional study involved a total of 288 hemodialysis patients with a confirmed diagnosis of ESRD (glomerular filtration rate ≤15 mL/min). Patients with a history of recurrent psychotic episodes, prior psychiatric illness (depression, generalized anxiety disorder, or severe mental illnesses), substance abuse, and a family history of psychiatric ailment were excluded from the study. The exclusion criteria were applied after appropriate history taking. All patients underwent evaluation for depression using the Patient Health Questionnaire-9 (PHQ-9) scale. Only patients with moderate-to-severe depressive symptoms on PHQ-9 were further evaluated for sleep quality and compliance to medications using the Pittsburgh Sleep Quality Index (PSQI) and Drug Attitude Inventory-10 (DAI-10), respectively. Patients with positive scores (from 0-10) on DAI-10 were labeled as compliant while negative scores were labeled as non-compliant. All patients were categorized into their relevant categories based on their PHQ-9, DAI-10, and PSQI scores. Patients with depression and poor sleep quality were referred to the psychiatry department for proper psychiatric consultation.

The normality of data was ascertained using the Kolmogorov-Smirnov test. The median scores of PHQ-9, DAI-10, and PSQI were calculated and baseline demographic variables were identified. Pertinently, the correlation of these study variables with PHQ-9 scores was calculated using Spearman’s rank-order correlation. Mann-Whitney U-test was used to identify the differences in PHQ-9 and PSQI scores among compliant and non-compliant patients. Next, we observed how PHQ-9, DAI-10, and PSQI scores were correlated using Spearman’s correlation matrix. A p-value of less than 0.05 was considered statistically significant. All statistical analysis was performed using Statistical Package for Social Sciences (SPSS, IBM, Armonk, NY, USA) version 25.0.

## Results

Out of the 288 patients, 188 (65.27%) had moderate-to-severe depressive illness, as identified using PHQ-9. A total of 114 patients were males while 74 were females. The baseline characteristics of the 188 study participants are shown in Table 1.

**Table 1:** Baseline characteristics of study participants.

Baseline characteristics		Frequency (n)	Percentages (%)
Gender	Males	114	60.6%
	Females	74	39.4%
Marital status	Married	147	78.2%
	Unmarried	41	21.8%
Employment status	Employed	133	70.7%
	Unemployed	55	29.3%
Dialysis session time	Morning	101	70.7%
	Evening	87	46.3%
Comorbidities	Diabetes mellitus	72	38.3%
	Ischemic heart disease	47	25%
	Hypertension	57	30.3%
	Asthma	9	4.8%
	No comorbid conditions	3	1.6%

The normality analysis revealed that the data were not normally distributed (p < 0.05). The median age of the study participants was 55 years ranging from 24 to 83 years. The details of study participants along with median PHQ-9, DAI-10, and PSQI scores are elucidated in Table 2.

**Table 2:** A breakdown of median scores of quantitative study variables. PHQ-9: Patient Health Questionnaire-9; DAI-10: Drug Attitude Inventory-10; PSQI: Pittsburgh Sleep Quality Index

Variables	Median	Range
Age	55	24-83
Dialysis years	3	1-15
Disease years	5.5	1-25
Time between diagnosis and dialysis in years	1.5	1-7
PHQ-9 scores	14.5	10-26
DAI-10 scores	1	-6 to +8
PSQI scores	7	2-21

A total of 113 (60.01%) of the depressed patients had poor compliance to medications while 137 (72.87%) patients had poor sleep quality. The breakdown of study participants with various PHQ-9, DAI-10, and PSQI categories are delineated in Table 3.

**Table 3:** An elucidation of PSQI, DAI-10, and PHQ-9 categories. PHQ-9: Patient Health Questionnaire-9; DAI-10: Drug Attitude Inventory-10; PSQI: Pittsburgh Sleep Quality Index

Variables		Frequency (n)	Percentages (%)
PSQI categories	Good sleep quality	51	27.1%
	Poor sleep quality	137	72.9%
DAI-10 categories	Compliant	75	39.9%
	Non-compliant	113	60.1%
PHQ-9 categories	Moderate depression	94	50%
	Moderately severe depression	66	35.1%
	Severe depression	28	14.9%

PHQ-9 scores were positively correlated with disease duration, dialysis years, and time between diagnosis and dialysis (r = 0.41, 0.39, and 0.43, respectively) and negatively with marital and employment status (r = -0.32 and -0.49, respectively). The Spearman’s correlation values of demographic variables with PHQ-9 and DAI-10 are shown in Table 4.

**Table 4:** Correlation of PHQ-9 and DAI-10 with study variables. PHQ-9: Patient Health Questionnaire-9; DAI-10: Drug Attitude Inventory-10

Parameters	Spearman’s correlation (r) with PHQ-9 scores	P-Value	Spearman’s correlation with DAI-10 scores	P-Value
Age	0.13	0.071	0.27	0.037
Disease duration (years)	0.41	0.004	-0.37	0.007
Dialysis years	0.39	0.015	-0.11	0.031
Time between diagnosis and dialysis	0.43	0.001	-0.28	0.024
Employed	-0.49	0.001	0.44	0.001
Married	-0.32	0.020	0.37	0.008

Non-compliant patients had significantly higher median PHQ-9 scores than compliant patients (p = 0.002). Furthermore, patients with poor sleep quality had higher PHQ-9 scores than those with good sleep quality (p = 0.017). This comparison is shown in Table 5.

**Table 5:** Median PHQ-9 scores across DAI-10 and PSQI categories. PHQ-9: Patient Health Questionnaire-9; DAI-10: Drug Attitude Inventory-10; PSQI: Pittsburgh Sleep Quality Index

Parameter	DAI-10 categories		P-Value (Mann-Whitney U-test)	PSQI categories		P-Value (Mann-Whitney U-test)
	Compliant	Non-compliant		Good sleep	Poor sleep	
Median PHQ-9 score (range)	11.5 (10-18)	17.5 (13-25)	0.001	12 (10-17)	17 (15-26)	0.004

Spearman’s correlation matrix showed that PHQ-9 scores were negatively correlated with DAI-10 but positively correlated with PSQI scores (Table 6).

**Table 6:** Spearman’s correlation matrix. PHQ-9: Patient Health Questionnaire-9; DAI-10: Drug Attitude Inventory-10; PSQI: Pittsburgh Sleep Quality Index

Parameters	PHQ-9 scores (Spearman’s rho, p-value)	PSQI scores (Spearman’s rho, p-value)	DAI-10 scores (Spearman’s rho, p-value)
PHQ-9 scores	1	0.61, 0.002	-0.54, <0.001
PSQI scores	0.61, 0.002	1	-0.58, <0.001
DAI-10 scores	-0.54, <0.001	-0.58, <0.001	1

## Discussion

The results of our study indicate that longer duration of disease, unemployment, not being married, and a shorter time between diagnosis and hemodialysis are significantly correlated with depression (p < 0.05). These associations are also supported in studies that identify similar factors [6,15]. A meta-analysis from Iran supports our finding of a high prevalence of depression indicating that nearly 57% of ESRD patients have depression [16]. However, in a study conducted in Texas, United States, the investigators found that the prevalence of depression in ESRD was 21% [17]. The difference in prevalence might be due to timely diagnosis and better psychiatric care in developed countries. On the contrary, poor psychiatric care, a low psychiatric workforce, and lack of physician’s awareness regarding the psychiatric components of ESRD lead to an increased prevalence of depression in developing countries [1-3]. The difference in these statistics indicates the immediate need for interventions to decrease the onset of depression in these patients.

Our study also indicates a positive correlation between PHQ-9 and PSQI scores and a negative correlation with DAI-10 scores. The higher scores on PSQI indicate poor sleep quality [18], indicating that increased severity of depression (higher PHQ-9 scores) translates into poorer sleep quality. However, the increased DAI-10 scores show more adherence to medications, which translates into poor compliance among patients with higher PHQ-9 scores. Studies have shown that depression decreases a patient’s motivation to take medicines, which explains the negative correlation of both variables in our study [18,19]. Sleep quality is important in maintaining adequate daytime functioning and poor sleep leads to daytime irritability and drowsiness decreasing the patient’s quality of life [19-21]. Poor sleep quality has also been associated with a higher mortality rate indicating its importance in disease prognosis [20]. Thus, there is an urgent need for timely assessment of sleep quality among ESRD patients so that the morbidity and mortality associated with the disease can be decreased with timely intervention.

A study highlighting the factors leading to depression in ESRD reported that social stigmatization and disability due to the chronic disease are important factors that contribute to the development of depression among ESRD patients [21]. Financial instability and fear of death might also play a role in the progression of depression in ESRD [19,22]. Identification of these concerns after the diagnosis of depressed patients will enable the physicians to point out the potential reasons for depression among them. Proper counseling regarding these concerns can help to alleviate depression in such patients so that they remain compliant with their medical therapy and better health care is provided to them. The prompt diagnosis and management of depression are important as they negatively impact the quality of life and physical well-being [3,14,22]. These effects coupled with decreased compliance and poor sleep quality worsen the disease prognosis and increase the mortality in hemodialysis patients [18,19]. This indicates the complex interplay among various factors affecting compliance and sleep among hemodialysis patients.

Unfortunately, the psychiatric components of the disease are not given adequate importance and are overlooked. Moreover, the lack of psychiatric consultation further deteriorates the mental health status of patients and along with the burden of ESRD, predisposes them to a depressive mental state. The results of our study necessitate proper attention to these psychiatric illnesses by clinicians so that these patients do not develop non-compliance or poor sleep quality. Furthermore, the use of non-pharmacologic interventions with cognitive behavioral therapy can also be helpful in treating such patients. Thus, the study highlights potential target areas that can be used to optimize medical therapy and improve the quality of life of hemodialysis patients. We recommend further case-control or cohort studies to evaluate the risk factors for depression so that the high-risk population can be identified and promptly treated using pharmacologic or non-pharmacologic interventions.

The inclusion of only a single public sector dialysis center as the study setting constitutes one of the limitations of our study. A multi-centered study including multiple public sector hospitals would enable the researchers to describe the prevalence of depression and trends of compliance in hemodialysis patients in a more precise manner. Other public health hospitals should be used to corroborate our study findings. Assessing the causes of depressive illness among the patients would have made our results more comprehensive. Moreover, the cultural aspects of the patients were not considered which accounts for another limitation in our study. Nonetheless, the results of our study necessitate the need for curation of specific guidelines that consider the psychiatric components of the disease for optimal multidisciplinary management.

## Conclusions

This study indicated an overall high prevalence of depression among hemodialysis patients. Non-compliance to medications and poor sleep quality are immensely common entities among hemodialysis patients with concomitant depression. Prolonged disease duration, more years of dialysis, and being unmarried or unemployed predisposed patients to develop more severe depression and poorer compliance to medications. The severity of depression is also associated with poor sleep quality and non-compliance among patients. The interplay of psychiatric components is complex in ESRD, and the interaction must be kept in mind so that optimal medical therapy can be offered to this subset of patients. Further cohort or case-control studies will help to evaluate the significant risk factors for depression in hemodialysis patients.
